# 4,4′-Bis[(*E*)-(2,3-diiodo­prop-2-en-1-yl)­oxy]biphen­yl

**DOI:** 10.1107/S1600536811003874

**Published:** 2011-02-05

**Authors:** Kiramat Shah, M. Raza Shah, Seik Weng Ng

**Affiliations:** a H.E.J. Research Institute of Chemistry, International Center for Chemical and Biological Sciences, University of Karachi, Karachi 7527, Pakistan; b Department of Chemistry, University of Malaya, 50603 Kuala Lumpur, Malaysia

## Abstract

Iodine adds across both triple bonds of 4,4′-bis­(prop-2-yn­­yl­oxy)biphenyl, yielding the 4,4′-bis­(2,3-diiodo­all­yloxy)biphenyl title compound, C_18_H_14_I_4_O_2_; the 2,3-diiodo­ally­oxy substituents have the I atoms in an *E* configuration. In the biphenyl portion of the mol­ecule, the aromatic rings are inclined by 37.8 (2)°.

## Related literature

For the structure of 4,4′-bis­(prop-2-yn­yloxy)biphenyl, see: Zhang *et al.* (2008 ▶). 
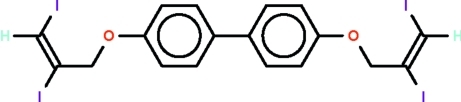


## Experimental

### 

#### Crystal data

C_18_H_14_I_4_O_2_
*M* *_r_* = 769.89
Triclinic, 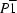

*a* = 10.0470 (4) Å
*b* = 10.2267 (4) Å
*c* = 11.3581 (4) Å
α = 105.760 (4)°
β = 101.433 (3)°
γ = 108.211 (4)°
*V* = 1014.45 (7) Å^3^
*Z* = 2
Mo *K*α radiation
μ = 6.15 mm^−1^
*T* = 100 K
0.20 × 0.10 × 0.05 mm

#### Data collection

Agilent SuperNova Dual diffractometer with an Atlas detector
Absorption correction: multi-scan (*CrysAlis PRO*; Agilent, 2010 ▶) *T*_min_ = 0.467, *T*_max_ = 1.000
8121 measured reflections
4502 independent reflections
4118 reflections with *I* > 2σ(*I*)
*R*_int_ = 0.026

#### Refinement

*R*[*F* ^2^ > 2σ(*F* ^2^)] = 0.030
*wR*(*F* ^2^) = 0.079
*S* = 1.04
4502 reflections
217 parameters
H-atom parameters constrained
Δρ_max_ = 3.38 e Å^−3^
Δρ_min_ = −1.69 e Å^−3^

### 

Data collection: *CrysAlis PRO* (Agilent, 2010 ▶); cell refinement: *CrysAlis PRO*; data reduction: *CrysAlis PRO*; program(s) used to solve structure: *SHELXS97* (Sheldrick, 2008 ▶); program(s) used to refine structure: *SHELXL97* (Sheldrick, 2008 ▶); molecular graphics: *X-SEED* (Barbour, 2001 ▶); software used to prepare material for publication: *publCIF* (Westrip, 2010 ▶).

## Supplementary Material

Crystal structure: contains datablocks global, I. DOI: 10.1107/S1600536811003874/jh2264sup1.cif

Structure factors: contains datablocks I. DOI: 10.1107/S1600536811003874/jh2264Isup2.hkl

Additional supplementary materials:  crystallographic information; 3D view; checkCIF report

## Figures and Tables

**Fig. 1.**
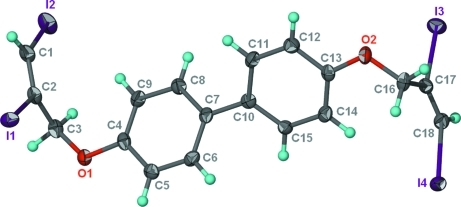
Thermal ellipsoid plot (Barbour, 2001) of C18H14I4O4 at the 70% probability level; hydrogen atoms are drawn as spheres of arbitrary radius.

## supplementary crystallographic information

### Comment

We have intended to activate the triple bond of 4,4'-bis(prop-2-ynyloxy)biphenyl (Zhang *et al.*, 2008) in order to polymerize the compound by using a cuprous iodide/iodine catalytic system. The attempt yielded instead the iodinated title compound (Scheme I, Fig. 1). The aromatic rings are twisted 37.8 (2) °.

### Experimental

Potassium carbonate (3 g, 1 mmol) and biphenyl-4,4'-diol (1 g, 5.3 mmol) were dissolved in ethanol (30 ml). The solution was heated for 1 h. This was followed by the addition propargyl bromide (1.5 ml, 17 mmol). The mixture was heated for another 8 h. The solvent was removed and the residue dissolved in a mixture of water (30 ml) and dichloromethane (30 ml). The aqueous layer was extracted three times with dichloromethane. Slow evaporation of dichloromethane gave colorless crystals (70%) of 4,4'-bis(prop-2-ynyloxy)biphenyl. This compound (1 g, 3.8 mmol) was dissolved an ethanol solution of cuprous iodide and iodine in an attempt to activate the triple bond. Slow evaporation of the solvent yielded the iodinated product in 80% yield.

### Refinement

Carbon-bound H-atoms were placed in calculated positions [C—H 0.95 to 0.99 Å, *U*_iso_(H) 1.2*U*_eq_(C)] and were included in the refinement in the riding model approximation.

The final difference Fourier map had a peak at 0.96 Å from I2 and a hole at 0.60 Å from the same atom.

### Crystal data

**Table:**

C_18_H_14_I_4_O_2_	*Z* = 2
*M**_r_* = 769.89	*F*(000) = 700
Triclinic, *P*1	*D*_x_ = 2.520 Mg m^−^^3^
Hall symbol: -P 1	Mo *K*α radiation, λ = 0.71073 Å
*a* = 10.0470 (4) Å	Cell parameters from 5987 reflections
*b* = 10.2267 (4) Å	θ = 2.4–29.2°
*c* = 11.3581 (4) Å	µ = 6.15 mm^−^^1^
α = 105.760 (4)°	*T* = 100 K
β = 101.433 (3)°	Prism, colorless
γ = 108.211 (4)°	0.20 × 0.10 × 0.05 mm
*V* = 1014.45 (7) Å^3^	

### Data collection

**Table:**

Agilent SuperNova Dual diffractometer with an Atlas detector	4502 independent reflections
Radiation source: SuperNova (Mo) X-ray Source	4118 reflections with *I* > 2σ(*I*)
Mirror	*R*_int_ = 0.026
Detector resolution: 10.4041 pixels mm^-1^	θ_max_ = 27.5°, θ_min_ = 2.4°
ω scans	*h* = −12→9
Absorption correction: multi-scan (*CrysAlis PRO*; Agilent, 2010)	*k* = −13→13
*T*_min_ = 0.467, *T*_max_ = 1.000	*l* = −9→14
8121 measured reflections	

### Refinement

**Table:**

Refinement on *F*^2^	Primary atom site location: structure-invariant direct methods
Least-squares matrix: full	Secondary atom site location: difference Fourier map
*R*[*F*^2^ > 2σ(*F*^2^)] = 0.030	Hydrogen site location: inferred from neighbouring sites
*wR*(*F*^2^) = 0.079	H-atom parameters constrained
*S* = 1.04	*w* = 1/[σ^2^(*F*_o_^2^) + (0.0391*P*)^2^ + 2.8781*P*] where *P* = (*F*_o_^2^ + 2*F*_c_^2^)/3
4502 reflections	(Δ/σ)_max_ = 0.001
217 parameters	Δρ_max_ = 3.38 e Å^−^^3^
0 restraints	Δρ_min_ = −1.69 e Å^−^^3^

### Fractional atomic coordinates and isotropic or equivalent isotropic displacement parameters (Å^2^)

**Table:**

	*x*	*y*	*z*	*U*_iso_*/*U*_eq_	
I1	0.52321 (3)	0.89339 (3)	0.77411 (3)	0.02006 (8)	
I2	0.05784 (3)	0.47289 (3)	0.70014 (3)	0.02903 (10)	
I3	0.69630 (3)	−0.38925 (3)	0.56256 (3)	0.02370 (9)	
I4	1.15769 (3)	−0.03766 (3)	0.91544 (3)	0.02524 (9)	
O1	0.5470 (4)	0.7096 (3)	0.9645 (3)	0.0202 (6)	
O2	0.8521 (4)	−0.0703 (3)	0.5344 (3)	0.0200 (6)	
C1	0.2354 (5)	0.6460 (5)	0.6979 (5)	0.0207 (9)	
H1	0.2259	0.6735	0.6242	0.025*	
C2	0.3610 (5)	0.7193 (5)	0.7945 (4)	0.0181 (9)	
C3	0.3993 (5)	0.7013 (5)	0.9214 (4)	0.0194 (9)	
H3A	0.3862	0.7788	0.9867	0.023*	
H3B	0.3297	0.6046	0.9150	0.023*	
C4	0.5783 (5)	0.5922 (5)	0.9042 (4)	0.0177 (9)	
C5	0.7224 (5)	0.6043 (5)	0.9543 (4)	0.0182 (9)	
H5	0.7893	0.6881	1.0270	0.022*	
C6	0.7679 (5)	0.4940 (5)	0.8981 (4)	0.0184 (9)	
H6	0.8666	0.5044	0.9316	0.022*	
C7	0.6700 (5)	0.3677 (5)	0.7927 (4)	0.0167 (8)	
C8	0.5257 (5)	0.3563 (5)	0.7479 (4)	0.0193 (9)	
H8	0.4568	0.2704	0.6782	0.023*	
C9	0.4797 (5)	0.4667 (5)	0.8023 (4)	0.0194 (9)	
H9	0.3806	0.4559	0.7696	0.023*	
C10	0.7198 (5)	0.2529 (5)	0.7296 (4)	0.0160 (8)	
C11	0.6679 (5)	0.1820 (5)	0.5947 (4)	0.0189 (9)	
H11	0.5995	0.2077	0.5448	0.023*	
C12	0.7143 (5)	0.0756 (5)	0.5334 (4)	0.0191 (9)	
H12	0.6784	0.0294	0.4424	0.023*	
C13	0.8144 (5)	0.0367 (4)	0.6066 (4)	0.0163 (8)	
C14	0.8670 (5)	0.1041 (5)	0.7393 (4)	0.0181 (9)	
H14	0.9346	0.0775	0.7891	0.022*	
C15	0.8192 (5)	0.2116 (5)	0.7992 (4)	0.0172 (8)	
H15	0.8558	0.2579	0.8902	0.021*	
C16	0.9582 (5)	−0.1128 (5)	0.6013 (4)	0.0192 (9)	
H16A	1.0423	−0.0230	0.6631	0.023*	
H16B	0.9961	−0.1666	0.5386	0.023*	
C17	0.8964 (5)	−0.2085 (5)	0.6726 (4)	0.0172 (8)	
C18	0.9551 (5)	−0.1992 (5)	0.7908 (4)	0.0201 (9)	
H18	0.9018	−0.2705	0.8211	0.024*	

### Atomic displacement parameters (Å^2^)

**Table:**

	*U*^11^	*U*^22^	*U*^33^	*U*^12^	*U*^13^	*U*^23^
I1	0.01788 (15)	0.02009 (15)	0.01828 (15)	0.00200 (11)	0.00372 (11)	0.00864 (12)
I2	0.01896 (17)	0.01891 (16)	0.0441 (2)	0.00325 (12)	0.01327 (14)	0.00592 (14)
I3	0.01724 (16)	0.02002 (16)	0.02533 (16)	0.00179 (12)	0.00369 (12)	0.00383 (13)
I4	0.02077 (16)	0.02426 (17)	0.02344 (16)	0.01197 (13)	−0.00226 (12)	0.00046 (13)
O1	0.0246 (17)	0.0183 (15)	0.0189 (15)	0.0112 (13)	0.0053 (13)	0.0061 (13)
O2	0.0263 (17)	0.0199 (16)	0.0201 (15)	0.0149 (13)	0.0081 (13)	0.0091 (13)
C1	0.014 (2)	0.014 (2)	0.031 (2)	0.0025 (17)	0.0072 (18)	0.0052 (19)
C2	0.016 (2)	0.014 (2)	0.025 (2)	0.0055 (16)	0.0100 (18)	0.0060 (18)
C3	0.025 (2)	0.019 (2)	0.020 (2)	0.0116 (18)	0.0126 (18)	0.0093 (18)
C4	0.024 (2)	0.016 (2)	0.019 (2)	0.0100 (17)	0.0108 (18)	0.0092 (17)
C5	0.022 (2)	0.014 (2)	0.018 (2)	0.0051 (17)	0.0054 (17)	0.0076 (17)
C6	0.016 (2)	0.020 (2)	0.020 (2)	0.0060 (17)	0.0037 (17)	0.0105 (18)
C7	0.020 (2)	0.016 (2)	0.018 (2)	0.0073 (17)	0.0059 (17)	0.0100 (17)
C8	0.019 (2)	0.018 (2)	0.021 (2)	0.0078 (18)	0.0058 (17)	0.0058 (18)
C9	0.018 (2)	0.021 (2)	0.022 (2)	0.0095 (18)	0.0062 (18)	0.0090 (19)
C10	0.016 (2)	0.014 (2)	0.018 (2)	0.0027 (16)	0.0060 (16)	0.0086 (17)
C11	0.017 (2)	0.020 (2)	0.021 (2)	0.0087 (18)	0.0041 (17)	0.0096 (18)
C12	0.021 (2)	0.019 (2)	0.017 (2)	0.0074 (18)	0.0055 (17)	0.0070 (18)
C13	0.017 (2)	0.0123 (19)	0.022 (2)	0.0045 (16)	0.0078 (17)	0.0088 (17)
C14	0.019 (2)	0.016 (2)	0.021 (2)	0.0067 (17)	0.0061 (17)	0.0113 (18)
C15	0.016 (2)	0.014 (2)	0.018 (2)	0.0037 (16)	0.0021 (16)	0.0054 (17)
C16	0.020 (2)	0.019 (2)	0.025 (2)	0.0107 (18)	0.0099 (18)	0.0131 (19)
C17	0.014 (2)	0.014 (2)	0.021 (2)	0.0053 (16)	0.0051 (17)	0.0041 (17)
C18	0.019 (2)	0.017 (2)	0.023 (2)	0.0063 (17)	0.0042 (18)	0.0077 (18)

### Geometric parameters (Å, °)

**Table:**

I1—C2	2.107 (4)	C7—C10	1.482 (6)
I2—C1	2.094 (4)	C8—C9	1.389 (6)
I3—C17	2.109 (4)	C8—H8	0.9500
I4—C18	2.089 (5)	C9—H9	0.9500
O1—C4	1.372 (5)	C10—C15	1.390 (6)
O1—C3	1.435 (6)	C10—C11	1.410 (6)
O2—C13	1.381 (5)	C11—C12	1.386 (6)
O2—C16	1.432 (5)	C11—H11	0.9500
C1—C2	1.331 (7)	C12—C13	1.400 (6)
C1—H1	0.9500	C12—H12	0.9500
C2—C3	1.493 (6)	C13—C14	1.386 (6)
C3—H3A	0.9900	C14—C15	1.398 (6)
C3—H3B	0.9900	C14—H14	0.9500
C4—C9	1.383 (6)	C15—H15	0.9500
C4—C5	1.399 (6)	C16—C17	1.500 (6)
C5—C6	1.392 (6)	C16—H16A	0.9900
C5—H5	0.9500	C16—H16B	0.9900
C6—C7	1.403 (6)	C17—C18	1.319 (6)
C6—H6	0.9500	C18—H18	0.9500
C7—C8	1.395 (6)		
			
C4—O1—C3	118.0 (3)	C8—C9—H9	120.1
C13—O2—C16	117.6 (3)	C15—C10—C11	117.5 (4)
C2—C1—I2	123.3 (4)	C15—C10—C7	122.1 (4)
C2—C1—H1	118.3	C11—C10—C7	120.4 (4)
I2—C1—H1	118.3	C12—C11—C10	121.5 (4)
C1—C2—C3	128.2 (4)	C12—C11—H11	119.3
C1—C2—I1	117.0 (3)	C10—C11—H11	119.3
C3—C2—I1	114.7 (3)	C11—C12—C13	119.5 (4)
O1—C3—C2	113.8 (4)	C11—C12—H12	120.2
O1—C3—H3A	108.8	C13—C12—H12	120.2
C2—C3—H3A	108.8	C14—C13—O2	125.7 (4)
O1—C3—H3B	108.8	C14—C13—C12	120.3 (4)
C2—C3—H3B	108.8	O2—C13—C12	114.0 (4)
H3A—C3—H3B	107.7	C13—C14—C15	119.2 (4)
O1—C4—C9	125.3 (4)	C13—C14—H14	120.4
O1—C4—C5	115.2 (4)	C15—C14—H14	120.4
C9—C4—C5	119.5 (4)	C10—C15—C14	122.0 (4)
C6—C5—C4	120.2 (4)	C10—C15—H15	119.0
C6—C5—H5	119.9	C14—C15—H15	119.0
C4—C5—H5	119.9	O2—C16—C17	113.0 (4)
C5—C6—C7	120.9 (4)	O2—C16—H16A	109.0
C5—C6—H6	119.5	C17—C16—H16A	109.0
C7—C6—H6	119.5	O2—C16—H16B	109.0
C8—C7—C6	117.5 (4)	C17—C16—H16B	109.0
C8—C7—C10	121.5 (4)	H16A—C16—H16B	107.8
C6—C7—C10	121.0 (4)	C18—C17—C16	128.5 (4)
C9—C8—C7	122.0 (4)	C18—C17—I3	116.8 (3)
C9—C8—H8	119.0	C16—C17—I3	114.6 (3)
C7—C8—H8	119.0	C17—C18—I4	124.2 (4)
C4—C9—C8	119.8 (4)	C17—C18—H18	117.9
C4—C9—H9	120.1	I4—C18—H18	117.9
			
I2—C1—C2—C3	−3.1 (7)	C8—C7—C10—C11	37.3 (6)
I2—C1—C2—I1	−178.41 (19)	C6—C7—C10—C11	−141.2 (4)
C4—O1—C3—C2	−73.4 (5)	C15—C10—C11—C12	−0.3 (7)
C1—C2—C3—O1	139.5 (5)	C7—C10—C11—C12	179.2 (4)
I1—C2—C3—O1	−45.1 (4)	C10—C11—C12—C13	0.3 (7)
C3—O1—C4—C9	1.6 (6)	C16—O2—C13—C14	−2.1 (6)
C3—O1—C4—C5	−177.3 (4)	C16—O2—C13—C12	177.8 (4)
O1—C4—C5—C6	−177.9 (4)	C11—C12—C13—C14	−0.1 (7)
C9—C4—C5—C6	3.1 (6)	C11—C12—C13—O2	−179.9 (4)
C4—C5—C6—C7	−1.5 (7)	O2—C13—C14—C15	179.6 (4)
C5—C6—C7—C8	−0.9 (6)	C12—C13—C14—C15	−0.2 (6)
C5—C6—C7—C10	177.6 (4)	C11—C10—C15—C14	0.0 (6)
C6—C7—C8—C9	1.8 (7)	C7—C10—C15—C14	−179.5 (4)
C10—C7—C8—C9	−176.8 (4)	C13—C14—C15—C10	0.3 (7)
O1—C4—C9—C8	178.9 (4)	C13—O2—C16—C17	74.9 (5)
C5—C4—C9—C8	−2.3 (7)	O2—C16—C17—C18	−135.0 (5)
C7—C8—C9—C4	−0.2 (7)	O2—C16—C17—I3	49.6 (4)
C8—C7—C10—C15	−143.3 (4)	C16—C17—C18—I4	1.3 (7)
C6—C7—C10—C15	38.2 (6)	I3—C17—C18—I4	176.6 (2)
